# A comparison of five methods to predict genomic breeding values of dairy bulls from genome-wide SNP markers

**DOI:** 10.1186/1297-9686-41-56

**Published:** 2009-12-31

**Authors:** Gerhard Moser, Bruce Tier, Ron E Crump, Mehar S Khatkar, Herman W Raadsma

**Affiliations:** 1 The CRC for Innovative Dairy Products, Australia; 2 Bellbowrie, QLD 4070, Australia; 3 Animal Breeding and Genetics Unit, University of New England, Armidale NSW 2351, Australia; 4 ReproGen - Advanced Technologies in Animal Genetics and Reproduction, Faculty of Veterinary Science, University of Sydney, 425 Werombi Road, Camden NSW 2570, Australia

## Abstract

**Background:**

Genomic selection (GS) uses molecular breeding values (MBV) derived from dense markers across the entire genome for selection of young animals. The accuracy of MBV prediction is important for a successful application of GS. Recently, several methods have been proposed to estimate MBV. Initial simulation studies have shown that these methods can accurately predict MBV. In this study we compared the accuracies and possible bias of five different regression methods in an empirical application in dairy cattle.

**Methods:**

Genotypes of 7,372 SNP and highly accurate EBV of 1,945 dairy bulls were used to predict MBV for protein percentage (PPT) and a profit index (Australian Selection Index, ASI). Marker effects were estimated by least squares regression (FR-LS), Bayesian regression (Bayes-R), random regression best linear unbiased prediction (RR-BLUP), partial least squares regression (PLSR) and nonparametric support vector regression (SVR) in a training set of 1,239 bulls. Accuracy and bias of MBV prediction were calculated from cross-validation of the training set and tested against a test team of 706 young bulls.

**Results:**

For both traits, FR-LS using a subset of SNP was significantly less accurate than all other methods which used all SNP. Accuracies obtained by Bayes-R, RR-BLUP, PLSR and SVR were very similar for ASI (0.39-0.45) and for PPT (0.55-0.61). Overall, SVR gave the highest accuracy.

All methods resulted in biased MBV predictions for ASI, for PPT only RR-BLUP and SVR predictions were unbiased. A significant decrease in accuracy of prediction of ASI was seen in young test cohorts of bulls compared to the accuracy derived from cross-validation of the training set. This reduction was not apparent for PPT. Combining MBV predictions with pedigree based predictions gave 1.05 - 1.34 times higher accuracies compared to predictions based on pedigree alone. Some methods have largely different computational requirements, with PLSR and RR-BLUP requiring the least computing time.

**Conclusions:**

The four methods which use information from all SNP namely RR-BLUP, Bayes-R, PLSR and SVR generate similar accuracies of MBV prediction for genomic selection, and their use in the selection of immediate future generations in dairy cattle will be comparable. The use of FR-LS in genomic selection is not recommended.

## Background

Until recently, the use of molecular genetics in commercial applications of marker-assisted selection (MAS) have focused on the use of individual genes or a few quantitative trait loci (QTL) linked to markers [1,2]. With the exception of a few genes with relatively large effects such as DGAT [3] or FECB [4] most candidate genes or QTL capture only a very small proportion of the total genetic variance. Recent empirical genome-wide association (GWAS) studies using a high-density SNP technology in humans, (*e.g*. [5-7], mice [8] and cattle [9] suggest that complex traits are most likely affected by many genes with a small effect.

A dramatic change in terms of the use of genomic information to estimate the total genetic value for breeding animals, known as genomic selection (GS) or Genome Wide Selection (GWS) was predicted by Meuwissen *et al*. [10]. Using simulations, they showed that with a dense marker map covering all the chromosomes, it is possible to accurately estimate the breeding value of animals without information about their phenotype or that of close relatives. Genomic estimated breeding values (GEBV) can be calculated for both sexes at an early stage in life, and therefore GS can increase the profitability and accelerate genetic gain of dairy cattle breeding by reducing the generation interval and cost of proving bulls [11,12]. This is projected to restructure dairy cattle breeding schemes, many of which rely on progeny testing sires and the recording of hundreds of thousands and often millions of cows [12].

Whole-genome analyses require methods that are capable of handling cases where the number of marker variables greatly exceeds the number of individuals, and models are at risk of being over parameterized. Furthermore, inclusion of complex pedigrees in large animal breeding data sets may lead to population stratification and confounding of relatedness with gene or SNP effects [13]. A variety of methods have been suggested for the estimation of genomic breeding values. Meuwissen *et al*. [10] have compared a joint least squares estimation of individually significant haplotype effects with best linear unbiased prediction (BLUP) including all haplotypes and two Bayesian approaches similar to BLUP, but allowing for variation in the genetic variance accounted for by individual haplotype effects. Xu [14] has used a similar Bayesian approach but has estimated additive and dominance effects attributed to individual marker loci rather than haplotype effects. As pointed out by Gianola *et al*. [15] Bayesian regression methods, such as those by Meuwissen *et al*. [10], require some strong *a priori *assumptions. These authors have proposed non-parametric kernel regression with a BLUP model accounting for the residual polygenes. Initially, these methods for computation of genomic breeding values have been investigated by simulation studies which may not be realistic in empirical situations and it is unlikely that the models underlying these simulations reflect the complexity of biological systems. Recently, other methods have been attempted in GWS analyses, *e.g*. principal component regression [16], partial least squares regression [17,18], LARS [19], LASSO [20,21], and BLUP including a genomic relationship matrix [22].

In this study we compared the accuracies of five different regression methods for the computation of genomic prediction of genetic merit in an empirical application. The selection of methods was based on their inherent differences in the underlying assumptions and previous application in GS. We analyzed a data set of 7,372 SNP markers genotyped on 1,945 Australian Holstein Friesian dairy bulls with highly reliable estimated breeding values (EBV) derived from phenotypic records of large groups of progeny.

## Methods

### Statistical models

Five regression methods were used to estimate SNP effects: fixed regression using least squares (FR-LS), random regression BLUP (RR-BLUP), Bayesian regression (Bayes-R), partial least squares regression (PLSR); and support vector regression (SVR). Other than the requirement that markers are located across the genome, no additional information, such as marker location or pedigree, is required by the methods. The basic model can be denoted as

where *y*_*i *_is the estimated breeding value (EBV) of sire *i *(*i *= 1, 2,..., *n*) and **x**_*i *_is a 1 × *p *vector of SNP genotypes on bull *i*, and *g*(**x**_*i*_) is a function relating genotypes to EBVs and can be considered as a molecular breeding value (MBV) and *e*_*i *_is a residual term. The SNP genotypes are coded as variates according to the number of copies of one SNP allele, *i.e*. 0, 1 or 2. We denote with **X **the matrix containing the column vectors **x**_*k *_of SNP genotypes at locus *k *(*k *= 1, 2, ..., *p*).

#### Fixed regression-least squares (FR-LS)

In linear regression on SNP, *g*(**x**_*i*_) is modeled as

where *β*_*k *_is the regression of EBV on the additive effect of SNP *k*, and *q *the number of SNP fitted in the model. The multicollinearity between SNP, *i.e*. two or more SNP in high but not complete LD, is addressed by selecting a limited number of 'important' SNP. A stepwise procedure in which markers are considered for inclusion in the model one at a time was used, as applied by Habier *et al*. [13]. Each marker that is not already in the model is tested for inclusion in the model. In each step the most significant SNP which had a *P*-value below a predefined threshold *α *was added to the model. *P*-values of all markers in the current model were then checked and the marker with the highest *P*-value above *α *was dropped from the model. The procedure stopped when no further addition or deletion was possible. The optimal *P*-value was found by cross-validation.

#### Random regression-BLUP (RR-BLUP)

In RR-BLUP, SNP effects are assumed random [10], with *g*(**x**_*i*_) having the form

where *β*_*k *_is the effect associated with SNP *k*, **x**_*k *_is set up as described above for additive effects. The regression coefficients are found by solving the normal equations,

where *λ *is constant for all SNPs. Differences in shrinkage between SNP still arise as a result of variation in allele frequency. Meuwissen *et al*. [10] and Habier *et al*. [13] have calculated *λ *for their simulated data from known genetic and residual variances. With no knowledge of these variance components and analyzing EBV data, an appropriate value for the shrinkage parameter can be obtained by cross-validation. When EBV have a variety of reliabilities then the regression can be weighted accordingly so that , where **R **is a diagonal matrix of weights. In this case most reliabilities exceeded 0.85 so they were treated as homogeneous, *i.e*. **R **= **I**.

#### Bayesian regression (Bayes-R)

Bayesian regression on additive SNP effects was performed as proposed by Meuwissen *et al*. [10] for their method BayesA using a Gibbs Sampler. It differs from the RR-BLUP model in that each SNP effect has its own posterior distribution. This model allows each marker to have its own variance, resulting in different shrinkage of SNP effects. The prior of Meuwissen *et al*. [10] was used for SNP effects.

#### Support vector regression (SVR)

Support vector machines are algorithms developed from statistical learning theory. Support vector regression (SVR, Vapnik [23]) uses linear models to implement non-linear regression by mapping the input space to a higher dimensional feature space using kernel functions. A feature of SVR is that it simultaneously minimizes an objective function which includes both model complexity and the error on the training data. SVR can be considered as a specific learning algorithm for reproducing kernel Hilbert spaces (RKHS) regression, first proposed for whole-genome analysis of quantitative traits by Gianola *et al*. [15]. A precise account of the theory of RKHS and details of SVR is beyond the scope of this article, so only a brief description is given here. For essential theoretical details and term definitions we refer the reader to Gianola *et al*. [15], Gianola and van Kaam [24]), Gianola and de Los Campos [25] and de Los Campos *et al*. [26]. An application of the RKHS regression approach to estimate genetic merit for early mortality in broilers from SNP data is described in [27]. A more detailed introduction to SVR is given in [28].

RKHS regression estimation is based on minimization of the following functional (*e.g*. equation 2 in [26]):(1)

where *V*(*y*_*i*_, *g*(**x**_*i*_)) is some loss (error) function, the second term in the equation acts as an penalty, and *λ *is a fixed positive real number that somehow controls the trade-off between the two terms and ∥∥^2 ^denotes the norm under a Hilbert space. Several choices of the loss function *V *in (1) are possible [28]. In their application of RKHS regression [27], used *V*(*y*_*i*_, *g*(**x**_*i*_)) = (*y*_*i *_- *g*(**x**_*i*_))^2 ^(equation (1) in [27]) as the loss function, which corresponds to the conventional least squares error criterion. In SVR the quadratic error function is replaced by a function called epsilon-insensitive loss proposed by Vapnik [23]:

It can be shown that the minimizer of (1) using epsilon-insensitive loss can be written as:

*j *= 1, 2,..., *n*, where *K *= (**x**_*i*_, **x**_*j*_) is the kernel involving the genotypes of sires *i *and *j*. The coefficients *α*_*i *_and *α*_i_* are the solution of a system of nonlinear equations. The loss function assigns zero loss to errors less than *ε*, thus safeguarding against overfitting. The parameter *ε *also provides a sparse representation of the data as only a fraction of the coefficients *α*_*i*_, *α*_i_* are nonzero. Data points associated with non-zero coefficients are called support vectors and a detailed interpretation of support vectors is given in [23]. Unlike the case of the quadratic loss function, where the coefficients *α*_*i *_are found by solving a linear system, using epsilon-insensitive loss the coefficients *α*_*i *_are the solutions to a quadratic programming problem.

In our implementation of SVR we used a Gaussian kernel. In order to solve the SVR regression problem three meta-parameters must be specified; the insensitivity zone *ε*, a penalty parameter *C *> 0 that determines the trade-off between approximation error and the amount up to which deviations larger than *ε *are tolerated and the bandwidth of the kernel function. Cross validation employing a grid search was used to tune the meta-parameters.

#### Partial least squares regression (PLSR)

A dimension reduction procedure, partial least squares regression (PLSR, [29]), was used for modeling without imposing strong assumptions. The main idea of PLSR is to build orthogonal components (called 'latent components') from the original predictor matrix **X **and use them for prediction in place of the original variables. Thus, *g*(**x**_*i*_) can be expressed as:

where **t**_*a *_is latent component *a *(*a *= 1, 2..., *h*) and generally *h *<<*p*. PLSR is similar to the well-known principal component regression (PCR), both methods construct a matrix of latent components **T **as a linear transformation of **X**, **T **= **XW**, where **W **is a matrix of weights. The difference is that PCR extracts components that explain the variance of **X**, whereas PLSR extracts components that have a large covariance with **y**, *i.e*. the columns of weight matrix **W **are defined such that the squared sample covariance matrix between **y **and the latent components is maximized under the constraint that the latent components are mutually uncorrelated.

Different techniques to extract the latent components exist, and each gives rise to a variant of PLSR. We implemented PLSR using an algorithm by Dayal and MacGregor [30], which does not require the calculation of the sample covariance matrix of **X **and which we have used previously [17]. A different PLS algorithm was used by Solberg *et al*. [18] to predict genomic breeding values in their simulation study. The optimal model complexity (*i.e*. number of latent components) was estimated by cross-validation.

### Animals and SNP data

**Figure 1:**
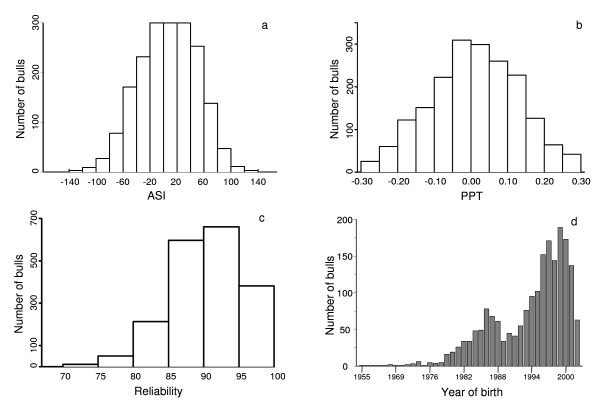
**Distribution of EBVs for Australian Selection Index (ASI, a) and protein percentage (PPT, b), distribution of reliabilities of EBVs (c), and number of bulls within year of birth (d)**.

A total of 1,945 progeny tested Holstein Friesian dairy bulls born between 1955 and 2002 were used in the study. The phenotypes used were Australian breeding values (EBV) taken from the August 2007 Australian Dairy Herd Improvement Scheme (ADHIS; http://www.adhis.com.au/) evaluation. The traits analyzed included protein percentage (PPT) and Australian Selection Index (ASI). ASI is a production based index that combines protein yield, fat yield and milk yield EBV and is weighted in relation to the value of the milk components: (ASI = 3.8 × protein EBV + 0.9 × fat EBV - 0.048 × milk EBV). The mean reliability of the EBV for both ASI and PPT was 0.89, with corresponding distribution of variation in range of reliability shown in Figure 1c. The distribution of EBV for both ASI and PPT for all 1,945 bulls is shown in Figure 1a and 1b, respectively.

The genotypic data belonged to a panel of 1,546 bulls genotyped for a 15K SNP chip and of 441 bulls for a GeneChip^® ^Bovine Mapping 25K SNP chip http://www.affymetrix.com/. There were 9,217 SNP and 44 bulls in common between these two datasets. A combined data set on 7,372 common SNP were extracted for the present study after removing SNP with low minor allele frequency (< 0.01), with low call rates (<80%), that deviated from Hardy-Weinberg equilibrium (*P *≤ 0.0001) or which showed inconsistent inheritance [31]. The proportion of missing SNP genotypes was less than 1%. We performed genotype imputation using the NIPALS (nonlinear iterative partial least squares, [32]) algorithm, which performs principal component analysis in the presence of missing data.

### Partitioning the data in training and test data sets

To assess the ability to predict breeding values of young bulls based on SNP data before progeny data were available, animals born before 1998 (N = 1,239) were included in the training set. Bulls born between 1998 and 2002 represented five single year cohorts and were allocated to test sets according to their year of birth.

### Model optimization

Applying FR-LS, RR-BLUP, SVR and PLSR requires the selection of appropriate meta-parameters. Model optimization was performed by 5-fold cross-validation. The complete training set (N = 1,239) was partitioned in K = 5 folds. For a given value of the meta-parameter(s) *θ *the prediction model is estimated using K-1 folds, and the predictive capacity of the model is assessed by applying the estimated model to the individuals in the left-out fold and this process is repeated K times so that every fold is left out once. The value of *θ *which minimized the average mean squared error of prediction (MSEP) in the K test sets was then used in the model to estimate the SNP effects from the full training set.

### Accuracy and bias of MBV prediction

The correlation coefficient between the realized EBV and the predicted MBV (r_EBV,MBV_) was used as a measure of the accuracy of MBV prediction. The realized EBV were linearly regressed on the predicted MBV, where the regression coefficient b_EBV,MBV _reflects the degree of bias of the MBV prediction. The interest here is the comparisons between bulls and therefore the constant estimated in the regression of EBV on MBV is of less interest and is not reported. The bias relates to the size of the absolute differences between MBV among cohorts, *i.e*. the estimate of the difference between a pair of bulls is greater (b_EBV,MBV _< 1) or less (b_EBV,MBV _> 1) than the difference between their EBV. A regression coefficient of one indicates no bias.

The MBV predictions of young bulls were combined with pedigree based predictions into an estimate of genomic estimated breeding values (GEBV) as GEBV = (w_1 _MBV + w_2 _SMGS)/(w_1 _+ w_2_), where SMGS are predictions based on the sire maternal-grandsire pathway and  with i = 1 for MBV and i = 2 for SMGS. For MBV, R^2 ^was calculated as the squared correlation between realized EBVs and MBV predictions (r_EBV,MBV_) from cross-validation of the training data. For SMGS, R^2 ^was calculated as the squared correlation between the realized EBV and SMGS predictions calculated at the time of the birth of the bull calves (r_EBV,SMGS_). As a measure of the accuracy of GEBV prediction we calculated the correlation between realized EBVs and GEBV predictions (r_EBV,GEBV_).

An analysis of variance was performed to investigate the effect of trait, method and test year on the accuracy and bias of MBV prediction. The regression coefficient was log_e_-transformed to account for non-normality and unstable variance. A single linear model was fitted to each of the metrics,

where *y *is either r_EBV,MBV _or log_e_b_EBV,MBV_, *μ *is a mean, *trait *is the effect of trait (PPT, ASI), *year *is the effect of test cohort (1998, 1999,...2002) including the 5-fold cross-validation set as a level of year; *method *is the effect of method (FR-LS, RR-BLUP, Bayes-R, PLSR, SVR), *trait.method*, *method.year*, and *trait.year *are two-way interactions between main effects; and *ε *is a random error.

### Implementation

For Bayes-R, the MCMC chain was run for 200,000 cycles with the first 50,000 samples discarded as burn in. Posterior estimates of SNP effects are based on 15,000 samples, drawing every 10^th ^sample after burn-in. The Gauss-Seidel algorithm with residual uptake suggested by Legarra and Misztal [33] was used in Bayes-R. FR-LS, PLSR, RR-BLUP and Bayes-R were implemented in Fortran, for SVR analyses we used the C++ library LIBSVM [34].

## Results

### Summary statistic on phenotypes

Despite the fact that most sires were pre-selected as young bulls on the basis of pedigree information, the distribution for both ASI and PPT is fairly symmetric (Figure 1a and 1b). One would not expect a noticeable genetic trend for PPT which was not part of the selection goal in the past. ASI was introduced in 1997 such that 63% of animals were born before the first ASI EBVs became available, ASI and was incorporated into a new profit-centered breeding objective in 2003.

The majority of the sires were test mated at approximately one year of age and their daughters subsequently brought into milk and performance tested. The body responsible for the genetic evaluation of dairy cattle in Australia publishes a single reliability value for all production traits and ASI. The published reliabilities of the EBV for ASI and PPT had an average of 0.89, with over 84% of animals having reliabilities of 0.85 or higher (Figure 1c). The age distribution of the genotyped bulls is shown in Figure 1d. Around 50% of the bulls were born after 1995, with a greater number of animals in the more recent cohorts.

### Model optimization, accuracy and bias of MBV prediction obtained by cross-validation

**Figure 2:**
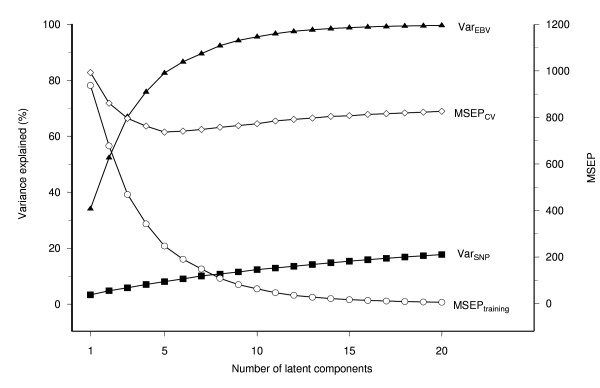
**Partial least squares regression model optimization for Australian Selection Index using cross-validation**. Shown is the mean prediction error (MSEP) in the training (MSEP_training_) data set, the average MSEP in the 5-fold cross-validation samples (MSEP_CV_), the proportion of EBV (Var_EBV_) and SNP variance (Var_SNP_) explained in the training data for models with an increasing number of latent components; the optimal prediction model includes the first 5 latent components, identified by the smallest MSEP_CV_.

The use of PLSR for genomic prediction has been described before [17,18] but we briefly illustrate the method by showing the cross-validation results for the analysis of ASI (Figure 2). A series of models with increasing numbers of latent components from 1 to 20 was fitted. The proportion of variance explained in the training samples shows that a small number of latent components provided an adequate fit of the data, with the first eight latent components explaining more than 90% of the EBV variance and the first latent component accounting for 34% alone. The prediction error in the corresponding test sets (MSEP_CV_) identified the first five latent components as having the lowest MSEP (Figure 2). The model with five latent components used only 8% of the SNP variance in the training set, suggesting a high degree of multicollinearity among SNP loci.

Table 1 provides a summary statistic of optimizing the threshold parameter *α *used to select SNP for the FR-LS prediction model by cross-validation. Shown are the mean accuracies obtained by predicting the five cross-validation samples, standard errors of accuracy and bias of prediction were computed from the variance of the means of the five cross-validation samples. As expected, the number of markers selected in the prediction model decreased with more stringent threshold values of *α*. The optimal model for ASI with the lowest MSEP was obtained with *α *= 0.001 including 33 markers, resulting in an accuracy of MBV prediction of 0.53. Similarly the best model for PPT was obtained at *α *= 0.001 (based on 30 SNP on average) with accuracy of prediction of 0.43, whereas the worst model was obtained at *α *= 0.1 (with inclusion of 215.6 SNP on average) with an accuracy of prediction of 0.35. In general the differences in accuracy of prediction between models including different number of SNP are small, and are small compared to their standard error. The degree of bias is assessed by comparing the regression coefficient of EBV on MBV with the value 1. The results show that for all α values predictions had a large bias as shown by a regression coefficient markedly less than 1 and bias decreased with decreasing number of SNPs.

**Table 1:** Cross-validation results for method fixed regression-least squares at different threshold values

Trait	*α*^†^	nSNP		MSEP		r_EBV,MBV_		b_EBV,MBV_	
ASI	0.1	197.2	(31.6)	1464	(139.2)	0.52	(0.031)	0.49	(0.059)
	0.01	98.2	(7.1)	1235	(62.1)	0.54	(0.043)	0.58	(0.045)
	0.001	33.0	(5.4)	1090	(124.4)	0.53	(0.036)	0.71	(0.048)
	0.0001	15.0	(1.9)	1108	(136.8)	0.50	(0.043)	0.76	(0.084)
									
PPT	0.1	215.6	(29.3)	0.0214	(0.0023)	0.35	(0.056)	0.32	(0.059)
	0.01	81.6	(5.0)	0.0156	(0.0016)	0.42	(0.059)	0.48	(0.075)
	0.001	30.0	(4.2)	0.0135	(0.0023)	0.43	(0.089)	0.62	(0.155)
	0.0001	15.4	(2.1)	0.0136	(0.0016)	0.39	(0.076)	0.67	(0.173)

Average number of SNP (nSNP) in the model, mean square error (MSEP), correlation (r_EBV,MBV_) between EBV and MBV, and regression coefficient (b_EBV,MBV_) of EBV on MBV for different threshold levels (*α*) in five cross-validation samples of the training data set, standard error in parentheses; ASI: Australian Selection Index; PPT: protein percentage; ^† ^*P*-value used to select SNPs in or out of model.

Table 2 summarizes the results for the various methods obtained from cross-validation of the training set. Accuracies of prediction ranged from 0.53 to 0.72 for ASI and 0.43 to 0.58 for PPT, respectively. All methods that used the information of all 7,372 SNP outperformed FR-LS. Accuracies of prediction and prediction errors for methods that estimate effects of all markers were essentially the same, although for ASI and PPT, SVR had the lowest MSEP, and the highest accuracy of prediction of 0.72 and 0.58, respectively. For both traits predictions obtained by FR-LS and PLSR had the largest bias, whereas for RR-BLUP, SVR and Bayes-R the regression of EBV on MBV was close to one.

**Table 2:** Summary of MBV prediction in the training data for five methods obtained by cross-validation

Trait	Method	MSEP		r_EBV,MBV_		b_EBV,MBV_	
ASI	FR-LS	1,090	(124.4)	0.53	(0.036)	0.71	(0.048)
	RR-BLUP	712	(93.5)	0.71	(0.017)	1.07	(0.076)
	Bayes-R	714	(95.3)	0.71	(0.016)	1.09	(0.071)
	SVR	700	(92.2)	0.72	(0.017)	1.06	(0.079)
	PLSR	735	(95.4)	0.70	(0.022)	0.93	(0.069)
							
PPT	FR-LS	0.0135	(0.0023)	0.43	(0.089)	0.62	(0.155)
	RR-BLUP	0.0104	(0.0018)	0.56	(0.067)	1.01	(0.104)
	Bayes-R	0.0104	(0.0010)	0.56	(0.067)	1.06	(0.117)
	SVR	0.0100	(0.0010)	0.58	(0.064)	1.01	(0.100)
	PLSR	0.0109	(0.0012)	0.55	(0.061)	0.81	(0.078)

Mean square error (MSEP), correlation (r_EBV,MBV_) between EBV and MBV, and regression coefficient (b_EBV,MBV_) of EBV on MBV derived by 5-fold cross-validation of the training data set, standard errors in parentheses; ASI: Australian Selection Index; PPT: protein percentage; FR-LS: fixed regression-least squares; RR-BLUP: random regression-BLUP; Bayes-R: Bayesian regression; SVR: support vector regression; PLSR: partial least squares regression.

### Accuracies in young bull cohorts

Table 3 shows the accuracy of MBV prediction of young bulls according to their year of birth and of the total test sample containing 706 animals. Marker effects were estimated using all animals in the training set with the best model of each method obtained by cross-validation. The FR-LS models included 48 SNP for ASI and 29 SNP for PPT. For ASI, accuracies of prediction (r_EBV,MBV_) of all young bulls were 0.45, 0.42, 0.41, 0.39, 0.27 for SVR, PLSR, Bayes-R, RR-BLUP and FR-LS, respectively. As for ASI, accuracies of MBV prediction of PPT were very similar between methods that used all SNP information (r_EBV,MBV _= 0.55-0.61), whereas FR-LS was the least accurate method (r_EBV,MBV _= 0.47).

**Table 3:** Correlation (r_EBV,MBV_) between EBV and MBV and regression coefficient (b_EBV,MBV_) of EBV on MBV in cohorts of young bulls for five methods

Trait	Method	Year of birth					
		
		1998	1999	2000	2001	2002	1998-2002
r_EBV,MBV_							
ASI	FR-LS	0.22	0.23	0.33	0.26	0.12	0.27
	RR-BLUP	0.35	0.39	0.40	0.32	0.28	0.39
	Bayes-R	0.38	0.38	0.42	0.33	0.29	0.41
	SVR	0.42	0.40	0.46	0.40	0.35	0.45
	PLSR	0.39	0.38	0.40	0.35	0.34	0.42
							
PPT	FR-LS	0.48	0.52	0.41	0.46	0.43	0.47
	RR-BLUP	0.53	0.58	0.53	0.56	0.49	0.55
	Bayes-R	0.63	0.60	0.55	0.63	0.51	0.60
	SVR	0.64	0.61	0.57	0.63	0.52	0.61
	PLSR	0.63	0.55	0.50	0.62	0.43	0.56
							
b_EBV,MBV_							
ASI	FR-LS	0.18	0.25	0.29	0.26	0.11	0.26
	RR-BLUP	0.59	0.72	0.82	0.72	0.60	0.72
	Bayes-R	0.59	0.71	0.81	0.71	0.59	0.76
	SVR	0.61	0.65	0.76	0.77	0.66	0.74
	PLSR	0.45	0.49	0.55	0.51	0.48	0.55
							
PPT	FR-LS	0.58	0.62	0.45	0.59	0.40	0.55
	RR-BLUP	1.09	0.93	0.98	1.10	0.72	1.08
	Bayes-R	1.24	1.10	1.15	1.29	0.81	1.16
	SVR	1.13	0.99	1.07	1.17	0.72	1.05
	PLSR	0.88	0.73	0.74	0.93	0.50	0.80
							

Number of bulls							
		144	189	173	137	63	706

The training data set included animals born before 1998; ASI: Australian Selection Index; PPT: protein percentage; FR-LS: fixed regression-least squares; RR-BLUP: random regression-BLUP; Bayes-R: Bayesian regression; SVR: support vector regression; PLSR: partial least squares regression.

In general the MBV predictions of PPT showed lower bias compared to those of ASI. MBV predictions of ASI obtained by all methods resulted in inflated differences in the relative rankings of bulls compared to relative rankings based on EBV. For PPT, predictions obtained by RR-BLUP and SVR were close to being unbiased compared to predictions of MBV obtained by PLSR and Bayes-R.

**Figure 3:**
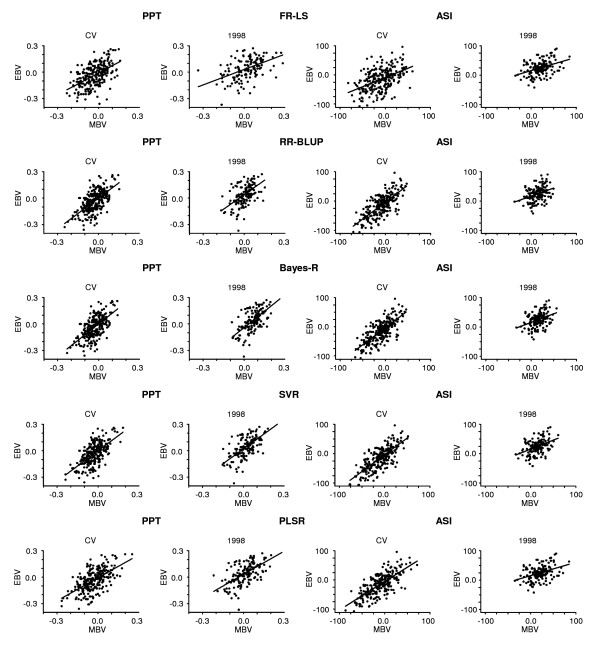
**Fit of models relating EBVs and predicted MBVs in the training data and in young bulls**. To avoid cluttering predictions are plotted for a single fold of the cross-validation (CV) of the training data set and young bull cohort 1998; ASI: Australian Selection Index; PPT: protein percentage; FR-LS: fixed regression-least squares; RR-BLUP: random regression-BLUP; Bayes-R: Bayesian regression; SVR: support vector regression; PLSR: partial least squares regression.

Figure 3 shows fits relating MBV predictions and realized EBVs of ASI and PPT in a single cross-validation sample, and in a young bull cohort 1998 for each of the five methods. FR-LS showed a larger dispersion of MBV across the range of EBV in cohort 1998 for both traits compared to the other methods, which is consistent with the lower accuracy seen with this method.

Accuracies of MBV prediction of ASI in young bull cohorts were considerably lower compared to the accuracy obtained by cross-validation of the training set, whereas for PPT predictions of the test data set were as accurate as the predictions obtained from cross-validation. As depicted in Figure 3, the EBV variance for ASI in the test sample is much lower relative to the cross-validation sample, which can partly explain the decrease in accuracy of predictions of ASI in young bull cohorts.

### Comparison of methods for MBV prediction

Correlations between MBV predictions obtained by cross-validation of the training data set and the test data set of young bulls are shown in Table 4. Predictions obtained by FR-LS were considerably less similar (r = 0.72-0.83) to all other methods. Thus using a smaller number of SNP as fixed effects produces somewhat different predictions to methods which use all SNP. The correlations between methods that used all SNP information were very high (r>0.9) for both ASI and PPT.

**Table 4:** Pearson correlations of MBV predictions in the training data (above diagonal) and in cohorts of young bulls (below diagonal) between five methods

	**ASI**					**PPT**				
		
**Method**	**FR-LS**	**RR-BLUP**	**Bayes-R**	**SVR**	**PLSR**	**FR-LS**	**RR-BLUP**	**Bayes-R**	**SVR**	**PLSR**
	
FR-LS		0.73	0.74	0.73	0.71		0.66	0.67	0.66	0.64
RR-BLUP	0.57		1	0.99	0.97	0.59		1	0.98	0.97
Bayes-R	0.58	0.96		0.99	0.97	0.63	0.95		0.98	0.96
SVR	0.59	0.93	0.96		0.96	0.63	0.91	0.97		0.95
PLSR	0.55	0.93	0.97	0.95		0.60	0.92	0.97	0.93	

ASI: Australian Selection Index; PPT: protein percentage; FR-LS: fixed regression-least squares; RR-BLUP: random regression-BLUP; Bayes-R: Bayesian regression; SVR: support vector regression; PLSR: partial least squares regression.

**Figure 4:**
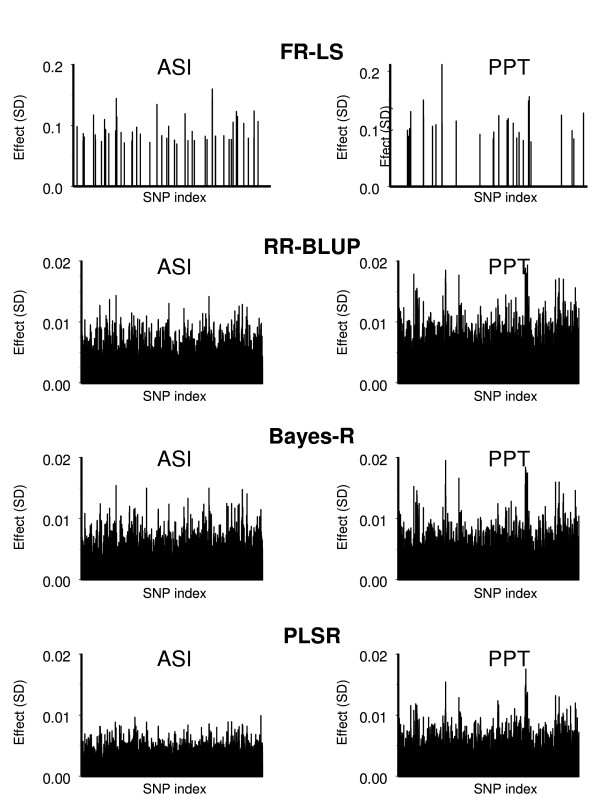
**Distribution of 7,372 SNP effects along the genome estimated by four methods**. The right most 772 SNPs are unassigned to chromosomes; ASI: Australian Selection Index; PPT: protein percentage; FR-LS: fixed regression-least squares; RR-BLUP: random regression-BLUP; Bayes-R: Bayesian regression.

Figure 4 shows the distribution of SNP effects along the genome estimated in the training data set for four methods for ASI and PPT. Relatively few SNP are used by the FR-LS method for both traits. The other methods assign relatively small effects to most of the SNP. However, the distribution for PPT depicts a small number of SNP with relatively large effects. All methods displayed very similar clustering of SNP with large effects along the genome.

### Increasing the number of bulls in the training data set

The accuracy of genomic predictions depends on the number of animals that are used to estimate the SNP effects [35]. The accuracy of MBV prediction estimated by PLSR from training data sets of increasing size are shown in Table 5. Larger training data sets did not result in significant gains in accuracy of MBV prediction in year cohorts of young bulls. In all cases predictions of PPT were more accurate than of ASI.

**Table 5:** Correlation (r_EBV,MBV_) between EBV and MBV in cohorts of young bulls with increasing size of the training data

	Training		r_EBV,MBV_				
		
	Year		1998	1999	2000	2001	2002
	
Trait		N	144	189	173	137	63
ASI	≤1997	1,239	0.39	0.38	0.40	0.35	0.34
	≤1998	1,383		0.37	0.38	0.29	0.26
	≤1999	1,572			0.45	0.35	0.30
	≤2000	1,745				0.39	0.34
	≤2001	1,882					0.32
							
PPT	≤1997	1,239	0.63	0.55	0.50	0.62	0.43
	≤1998	1,383		0.55	0.51	0.64	0.41
	≤1999	1,572			0.52	0.66	0.43
	≤2000	1,745				0.68	0.46
	≤2001	1,882					0.47

Results were obtained by cross-validation using partial least squares regression; ASI: Australian Selection Index; PPT: protein percentage

### Combining MBV and SMGS predictions

GEBV predictions for bulls born between 1998 and 2002 were calculated by combining the MBV predictions with the sire maternal-grandsire pathway predictions, which were calculated at the time of birth of the young bull calves (Table 6). The accuracy of GEBV prediction of ASI was 1.06 - 1.34 times higher than the accuracy of the pedigree based prediction, and 1.16 - 1.27 times higher for PPT. Among the methods FR-LS had the lowest accuracy and the differences between the other methods were small.

**Table 6:** Correlation (r_EBV,SMGS_) between EBV and pre-progeny test sire maternal-grandsire EBV prediction and correlation (r_EBV,GEBV_) between EBV and GEBV in young bulls for five methods

		r_EBV,GEBV_				
		
Trait	r_EBV,SMGS_	FR-LS	RR-BLUP	Bayes-R	SVR	PLSR
ASI	0.35	0.37	0.45	0.45	0.47	0.45
PPT	0.49	0.57	0.60	0.62	0.60	0.62

GEBV predictions for bulls born between 1998 and 2002 were calculated by combining the MBV predictions with the sire maternal-grandsire pathway predictions, which were calculated at the time of birth of the young bull calves; ASI: Australian Selection Index; PPT: protein percentage; FR-LS: fixed regression-least squares; RR-BLUP: random regression-BLUP; Bayes-R: Bayesian regression; SVR: support vector regression; PLSR: partial least squares regression.

### Variability in accuracy and bias of MBV prediction

Abridged analysis of variance tables for the accuracy and bias of MBV prediction are shown in Table 7. The method and the interaction between trait and year showed significant effects on the accuracy of MBV prediction. Accuracies of prediction by FR-LS and SVR were significantly different from other methods, with SVR being the most and FR-LS the least accurate method (additional file 1). Accuracy of prediction obtained by cross-validation of the training data was significantly higher than the accuracy of prediction in cohort 2002. The interactions between method and trait and between trait and year showed significant effects on the regression of EBV on MBV.

**Table 7:** Summary of ANOVA of factors affecting correlation (r_EBV,MBV_) between EBV and MBV and regression coefficient (log_e_b_EBV,MBV_) of EBV on MBV

	r_EBV,MBV_		log_e_b_EBV,MBV_	
		
Model Term	*P*-value^†^	*F*-value	*P*-value^†^	*F*-value
Method	< 0.001	48.88	n.t.	88.09
Trait	n.t.	350.84	n.t.	131.35
Year	n.t.	61.68	n.t.	20.44
Method.Trait	0.198	1.66	0.002	6.18
Method.Year	0.827	0.65	0.346	1.20
Trait.Year	< 0.001	60.19	<0.001	10.73

Shown are the significance level (*P*-value) and *F*-value of each model term; the regression coefficient was log_e_-transformed to account for non-normality and unstable variance; ^† ^n.t. non-testable.

### Computing time

Computing time is important, particularly for cross-validation and implementation in practice which requires frequent re-estimation of breeding values. The computational demand of the various methods is shown in Table 8. The machine used for all calculations had a dual core Intel D 3.2 GHz CPU. The PLSR and RR-BLUP methods, took less than 1 min to calculate the marker effects for a single replicate of the training data. The requirements of Bayes-R are several orders of magnitude higher (~421 min). The computational burden for SVR lies in the grid search for the meta-parameters and also requires the computation of the kernel matrix in the prediction step. PLSR, RR-BLUP and SVR all scale well to larger number of SNPs.

**Table 8:** Computation times for estimation of SNP effects for five methods

FR-LS	RR-BLUP	Bayes-R	SVR	PLSR
~3 min	~22 s	~421 min	~4 min	~8 s

Results were obtained by calculating SNP effects for a single replicate of the training data; FR-LS: fixed regression-least squares; RR-BLUP: random regression-BLUP; Bayes-R: Bayesian regression; SVR: support vector regression; PLSR: partial least squares regression.

## Discussion

The concept of genomic selection was first raised over eight years ago [10], but it was not until the advent of high capacity genotyping platforms that empirical data sets became available in dairy cattle [36-40]. Initial reports on efficiencies and pros and cons of different statistical approaches for genomic selection have been conducted largely on simulated data sets (*e.g*. [10,13,15,18,41-44]) and to a lesser extent in real data (*e.g*. [19,35,45,46]. Simulation studies, although informative, are strongly dependent on the underlying assumptions, some of which may be biologically unrealistic or limited in their complexity. This paper describes the performance of five statistical approaches for the prediction of molecular and genomic breeding values using empirical data.

### Comparison of methods

The choice of methods evaluated here represent a range of methods proposed previously for the potential use in genomic selection including variable selection methods (FR-LS, [10,13,47], shrinkage methods (Bayes-R and BLUP, [10,13,14]); support vector learning methods (SVR, [15,43]) and dimension reduction methods (PLSR, [17,18]).

Methods for calculating genomic breeding values have to deal with the problem of multicollinearity and over-parameterization resulting from fitting many parameters to relatively small data sets. The FR-LS regression method, which exploits a reduced subset of selected SNP consistently had lower accuracy and a larger bias of prediction than the other methods. Bayes-R, RR-BLUP, SVR and PLSR which use all available SNP information performed remarkably similarly; even though these methods are very different from each other. The performance of all methods depends on one or more meta-parameters. For RR-BLUP, SVR and PLSR optimal values of the meta-parameters are found by minimizing the prediction error using cross-validation, potentially leading to more robust predictions than Bayes-R where the posterior estimates of SNP effects are greatly affected by the choice of the parameters in the prior distributions. Using the same priors as in [10] Bayes-R performed similar to the optimized models of RR-BLUP, SVR and PLSR. One reason why these priors performed well might be that the frequency distribution of estimated SNP effects for ASI and PPT somewhat resembles the frequency distribution of SNP effects underlying the simulation in [10] which was derived from published estimated QTL effects [48].

It appears that the gain in accuracy of RR-BLUP, Bayes-R, SVR and PLSR over FR-LS is related to the increased number of SNP included in the model. FR-LS fitted only 48 SNP for ASI and 29 SNP for PPT in the prediction equations and including more SNP did not result in higher accuracies in the cross-validation set (Table 1). It is well known that FR-LS estimates of a subset of SNP effects or QTL are biased upwards and that SNP selection methods perform poorly on multicollinear markers [49]. An advantage of the use of multicollinear SNP is that it can increase the accuracy of estimates of effects. SNPs in high LD define a larger segment of genome and the standard deviation of the estimated effect of the segment is reduced by a factor of  when the average of n SNP is used instead of a single SNP. This averaging takes place when many SNP in high linkage disequilibrium are used to construct a model. An analogy is encountered in QTL mapping, when QTL inference is related to the peak area of a QTL, rather than the peak height at a given position. In general, prediction is not seriously affected by multicollinearity as long as the correlational structure observed in the training sample persists in the prediction population, *e.g*. [50]. With close genetic relationships between bulls in the training and tests set, as was the case here, methods that fit more SNPs capture more of the genetic relationships which can in fact lead to an increase in accuracies as shown by Habier *et al*. [13]. For example, sons and grandsons of the 10 most popular ancestors accounted for 37.6% of bulls in the training data set and 32.7% of young bulls in the test data set.

As technology platforms advance it is possible to extend the density to many thousands of SNP genotypes per individual, possibly capturing all sources of genomic variation with entire genomes being sequenced per individual. Such technology platforms will only exacerbate the curse of dimensionality and computational burden of a method will become more important. In particular, PLSR and RR-BLUP are very fast methods. The use of methods based on Bayesian regression on the other hand, such as BayesA, might be prohibitive when the number of SNP is large. For SVR computing time does not depend on the number of SNP but rather on the animals that are genotyped.

The relevance of methods which focus on identifying a subset of the available SNP will remain high while the cost of dense chips is high. Although subset selection by FR-LS performed poorly in our study and therefore cannot be recommended, some authors reported similar or improved accuracy when using a pre-selected subset of SNP. In the study of Moser *et al*. [17] the selection was performed within the PLSR and in Gonzalez-Recio *et al*. [45] within a kernel regression framework. The use of SNP subset selection in genomic selection needs further testing. In the end, it may be of limited use if multiple traits require so many SNP that the cost of genotyping them is similar to the cost of a high density chip.

### Application of GS and variability in accuracy of prediction

A key issue in genomic selection is predicting genetic merit in young animals across a wide range of traits. In the case of dairy cattle breeding, this is particularly advantageous given the sex limited expression of most traits and the long generation interval due to relying on progeny testing to select superior replacement sires. The potential advantages of using genomic selection in breeding programs of dairy cattle have been demonstrated by Schaeffer [11] and König *et al*. [12]. Although the assumptions may not be met by the currently achieved accuracies of GEBV, the principles of reduced generation interval and increased accuracy of selection of young bulls at time of entry into progeny test all show substantial benefits from increase in genetic gain and reduced costs.

Genomic breeding values that combined the marker-based MBV with a pedigree based polygenic effect had higher accuracies than MBV or polygenic component alone, which is consistent with reports in dairy cattle [35,46], wheat and mice [21]. We show here that accuracies of GEBV were approximately 1.3 times larger than accuracies of sire maternal-grandsire pathway breeding values, which are currently used to select bull calves to enter progeny testing. Most of the bulls in our study belong to the same breeding program as the population used to estimate GEBV by Hayes *et al*. [35], but there was no overlap between the training sets between studies as their training set included animals born between 1998 and 2002, some of which are presumably part of our test sets. Although direct comparison of both studies is difficult since there were differences in the number of bulls in the training data and in the method used to calculate accuracies of GEBV, Hayes *et al*. [35] reported higher accuracies of GEBV for ASI than for PPT. It remains uncertain to what extent MBV predictions of the same trait derived from different populations, or even different reference populations within the same breed, are robust and warrants further examination.

Improvement of accuracies of genomic predictions are likely to benefit from substantially increased training sets as individual SNP effects are estimated with greater accuracy [35,46]. In contrast with observations by VanRaden *et al*. [46], an increase in accuracy of MBV was not apparent with an increased number of bulls in the training set (Table 5). This discrepancy may rest with the smaller range in the number of bulls in the training set in our data (1,239-1,882) compared with 1,151 to 3,576 used in [46].

Gains in accuracy are expected from increased SNP densities, as a larger proportion of the QTL variance is explained by markers and effects of QTL can be predicted across generations as shown in simulated data sets [10,51]. VanRaden *et al*. [46] have shown a consistent but small increase in the coefficient of determination for genomic prediction for North American Holstein bulls from 0.39 to 0.43 when the map density increased from 9,604 to 38,416 SNP. We would expect a similar relatively small increase of the accuracy of MBV prediction with greater SNP densities here. The reason is that many animals in our training and test data share DNA segments from a small number of sires and relatively few markers are required to trace the chromosome segments shared between related animals separated by only a few generations. To what extent increasing SNP densities improves the accuracy of genomic prediction in populations with low effective population size remains to be seen. Greater SNP densities may be required in more divergent populations or when individual animal phenotypes are analyzed instead of EBVs derived from progeny test data.

As noted above, markers used in the statistical model not only estimate QTL effects but also capture genetic relationships between individuals in the training data [13]. Habier *et al*. [13,41] and Zhong *et al*. [44] have demonstrated differences in the contribution of LD between marker and QTL and marker-based relatedness for different statistical methods. They show a gradual decline of accuracy of prediction for individuals which are removed several generations from the training data set. Habier *et al*. [13] have found that RR-BLUP is affected by genetic relationships to a larger degree than FR-LS, predicting a steeper decline in the accuracy for RR-BLUP in generations following training. Here, we did not observe a gradual decline in accuracy of prediction, in fact for ASI correlations for all methods were higher in animals born in year 2000 compared to animals born in year 1998. Small rank changes in the performance of the methods between test years did occur, but there was no strong evidence for different rates of decline of accuracies in later test years between methods. This is supported by the non-significant interaction between method and year from the ANOVA. In practice, it will be difficult to differentiate between improvements in the accuracy of prediction resulting from modelling relationships via SNP or from LD between SNP and QTL. It is still likely that a significant component of the gains of GS will reside with predicting relationships more accurately on the genome level either within families [41] or even across families. For industry applications it is feasible and most likely that prediction equations can be updated as information on new animals becomes available, and this will ensure a minimal lag between animals in the training set and the test set.

In practice, the accuracy of predictions of future outcomes needs to be assessed. The partitioning of the data depicts this situation with older bulls in the training data and younger bulls in the test data. Care must be taken when the accuracy of future predictions is evaluated by cross-validation of random subsets of the training data set. A significant decrease in accuracy of prediction of young bull cohorts was observed for ASI relative to the accuracy obtained by cross-validation of the training data set, whereas for PPT the reduction in accuracy was negligible (Tables 2 and 3). In general a decrease in the accuracy of MBV in young bull cohorts might be expected, since accuracies of realized EBV for early proofs are likely to be lower than the accuracies of realized EBV of the older bulls in the training data set. Initially, this would not explain the differences in accuracy of MBV of young bulls seen between ASI and PPT, as the body responsible for the genetic evaluation of dairy cattle in Australia publishes a single reliability value for all production traits and ASI. However, heritabilities for the ASI component traits (0.25) are lower than for PPT (0.40) and more training animals may be required to obtain accurate prediction equations for traits with lower heritability.

Another reason for a decrease in accuracy of prediction of younger bull cohorts may be a reduction in EBV variance in pre-selected bull teams. As shown in Figure 3, the variance of PPT and ASI (column 1 and 2, respectively) of the training animals is greater compared to the variance in one of the young bull cohorts (column 3 and 4, respectively). This is likely to have affected the accuracy of ASI, since ASI is a strong component of the multi-trait profit index on which young bulls are currently selected, whereas no pre-selection is likely to have occurred on PPT. Finally the genetic architecture underlying traits may also affect the robustness of accuracy of prediction from SNP data, since for PPT compared to ASI, fewer individual SNP with relatively large effect contributed to the prediction equations.

## Conclusions

Five regression methods proposed to calculate genomic breeding values have been empirically evaluated using data of 1,945 dairy bulls typed for 7,373 genome-wide SNP markers. From our evaluations a number of important observations can be made. Firstly, FR-LS based models included a small number of markers and had poor accuracy and large bias of prediction. Secondly, accuracies of MBV prediction obtained by methods that estimate effects of all SNP were remarkably similar, despite the different assumptions underlying the models. Thirdly, accuracies derived by cross-validation with random subsets of the training data are likely to overestimate the realized accuracies of future predictions for some traits in young bull cohorts. Combining marker and pedigree information increased the accuracy of prediction but the gain was different for the two traits investigated. Computational demand of a method is potentially important in implementing genomic selection in practice and was lowest for PLSR and RR-BLUP, and highest for Bayes-R.

## Competing interests

The authors declare that they have no competing interests.

## Authors' contributions

GM was the principal investigator in the design of the study and methods, participated in the statistical methods, carried out the statistical analysis and drafted the manuscript. BT and RC participated in the statistical methods, discussions and helped revise the manuscript. MSK participated in the analysis, had a principal role in data acquisition, assembly and data QC and contributed to the manuscript preparation. HWR was the project leader, contributed to project design, data acquisition, result interpretation and had a leading role in manuscript preparation. All authors read and approved the manuscript.

## Authors' information

AGBU is a joint venture of NSW Department of Primary Industry and University of New England.

## Supplementary Material

Additional file 1 **Tables showing model-based means from ANOVA of factors affecting correlation (r_EBV,MBV_) between EBV and MBV and regression coefficient (log_e_b_EBV,MBV_) of EBV on MBV**. The regression coefficient was log_e_-transformed to account for non-normality and unstable variance. Estimates with different superscript are significantly different at the 0.05 significance level. Click here for file
