# Supplementary material for: A comparison of five methods to predict genomic breeding values of dairy bulls from genome-wide SNP markers
Source: Genet Sel Evol. 2009 Dec 31;41(1):56. doi: 10.1186/1297-9686-41-56 (PMC2814805; doi:10.1186/1297-9686-41-56)
Supplement: Additional file 1 — Tables showing model-based means from ANOVA of factors affecting correlation (rEBV,MBV) between EBV and MBV and regression coefficient (logebEBV,MBV) of EBV on MBV. The regression coefficient was loge-transformed to account for non-normality and unstable variance. Estimates with different superscript are significantly different at the 0.05 significance level. [file 1297-9686-41-56-S1.DOC]

**Model-based means from ANOVA of factors affecting correlation (r_EBV,MBV_) between EBV and MBV and regression coefficient (log_e_b_EBV,MBV_) of EBV on MBV**

**Metric: r_EBV,MBV_**, Factor: Method

| FRLS | RR_BLUP | Bayes-R | SVR | PLSR |
| --- | --- | --- | --- | --- |
| 0.368 ^a^ | 0.475 ^b^ | 0.499 ^b^ | 0.525 ^c^ | 0.487 ^b^ |

**Metric: r_EBV,MBV_,** Factor: Trait.Year

| Trait.Year | CV | 1998 | 1999 | 2000 | 2001 | 2002 |
| --- | --- | --- | --- | --- | --- | --- |
| ASI | 0.674^h^ | 0.352^b^ | 0.356^b^ | 0.0402^c^ | 0.332^b^ | 0.276^a^ |
| PPT | 0.536^ef^ | 0.582^g^ | 0.572^fg^ | 0.512^de^ | 0.580^g^ | 0.476^d^ |

**Metric: log_e_b_EBV,MBV_**, Factor: Trait.Method

| Trait.Method | FRLS | RR_BLUP | Bayes-R | SVR | PLSR |
| --- | --- | --- | --- | --- | --- |
| ASI | -1.373^a^ | -0.304^c^ | -0.311^c^ | -0.303^c^ | -0.598^b^ |
| PPT | -0.624^b^ | -0.038^d^ | 0.0892^d^ | 0.003^d^ | -0.287^c^ |

**Metric: log_e_b_EBV,MBV_**, Factor: Trait.Year

| Trait.Year | CV | 1998 | 1999 | 2000 | 2001 | 2002 |
| --- | --- | --- | --- | --- | --- | --- |
| ASI | -0.041^d^ | -0.813^ab^ | -0.640^bc^ | -0.504^c^ | -0.591^c^ | -0.879^a^ |
| PPT | -0.122^d^ | -0.050^d^ | -0.156^d^ | -0.182^d^ | -0.019^d^ | -0.495^c^ |

The regression coefficient was log_e_-transformed to account for non-normality and unstable variance. Estimates with different superscript are significantly different at the 0.05 significance level.
